# Reactive nitrogen species inhibit branched chain alpha-ketoacid dehydrogenase complex and impact muscle cell metabolism

**DOI:** 10.1016/j.jbc.2023.105333

**Published:** 2023-10-10

**Authors:** Nicholas L. Arp, Gretchen L. Seim, James A. Votava, Jordyn Josephson, Jing Fan

**Affiliations:** 1 Morgridge Institute for Research, Madison, Wisconsin, USA; 2 Cellular and Molecular Biology Graduate Program, University of Wisconsin-Madison, Madison, Wisconsin, USA; 3 University of Wisconsin Medical Scientist Training Program, University of Wisconsin School of Medicine and Public Health, Madison, Wisconsin, USA; 4 Department of Nutritional Sciences, University of Wisconsin-Madison, Madison, Wisconsin, USA

**Keywords:** α-ketoacid dehydrogenase, mitochondria metabolism, nitric oxide, metabolic regulation, branched chain amino acid

## Abstract

Branched chain α-ketoacid dehydrogenase complex (BCKDC) is the rate-limiting enzyme in branched chain amino acid (BCAA) catabolism, a metabolic pathway with great importance for human health. BCKDC belongs to the mitochondrial α-ketoacid dehydrogenase complex family, which also includes pyruvate dehydrogenase complex and oxoglutarate dehydrogenase complex. Here, we revealed that BCKDC can be substantially inhibited by reactive nitrogen species (RNS) *via* a mechanism similar to what we recently discovered with pyruvate dehydrogenase complex and oxoglutarate dehydrogenase complex—RNS can cause inactivating covalent modifications of the lipoic arm on its E2 subunit. In addition, we showed that such reaction between RNS and the lipoic arm of the E2 subunit can further promote inhibition of the E3 subunits of α-ketoacid dehydrogenase complexes. We examined the impacts of this RNS-mediated BCKDC inhibition in muscle cells, an important site of BCAA metabolism, and demonstrated that the nitric oxide production induced by cytokine stimulation leads to a strong inhibition of BCKDC activity and BCAA oxidation in myotubes and myoblasts. More broadly, nitric oxide production reduced the level of functional lipoic arms across the multiple α-ketoacid dehydrogenases and led to intracellular accumulation of their substrates (α-ketoacids), decrease of their products (acyl-CoAs), and a lower cellular energy charge. In sum, this work revealed a new mechanism for BCKDC regulation, demonstrated that RNS can generally inhibit all α-ketoacid dehydrogenases, which has broad physiological implications across multiple cell types, and elucidated the mechanistic connection between RNS-driven inhibitory modifications on the E2 and E3 subunits of α-ketoacid dehydrogenases.

The family of α-ketoacid dehydrogenase complexes, which includes pyruvate dehydrogenase complex (PDHC), oxoglutarate dehydrogenase complex (OGDC), and branched chain α-ketoacid dehydrogenase complex (BCKDC), play key roles in mitochondrial metabolism. These enzyme complexes comprise of three subunits and share a similar catalytic mechanism (Fig. 1*A*). The E1 subunit, PDH, OGDH, and BCKDH for the three enzyme complexes, respectively, is a thiamin-dependent decarboxylase that decarboxylates its substrate (α-ketoacids) to its corresponding acyl group. The E2 subunits, dihydrolipoamide S-acetyltransferase (DLAT), dihydrolipoamide S-succinyltransferase (DLST), and dihydrolipoamide branched chain transacylase (DBT), respectively, contain a covalently attached lipoic arm that mediates the transfer of the acyl group from the E1 subunit to CoA to produce acyl-CoA. With this transfer, the lipoic arm converts from its oxidized form (lipoamide) to its reduced form (dihydrolipoamide). Finally, the E3 subunit, dihydrolipoamide dehydrogenase (DLD), encoded by the same gene for all three α-ketoacid dehydrogenase complexes, re-oxidizes the dihydrolipoamide to lipoamide, coupled to NAD reduction to NADH. Together, the coupled action of the three subunits allows for the oxidation of α-ketoacids and the production of acyl-CoA and NADH. These enzymes catalyze reactions that are key steps in carbohydrates and amino acids catabolism (1).

**Figure 1:**
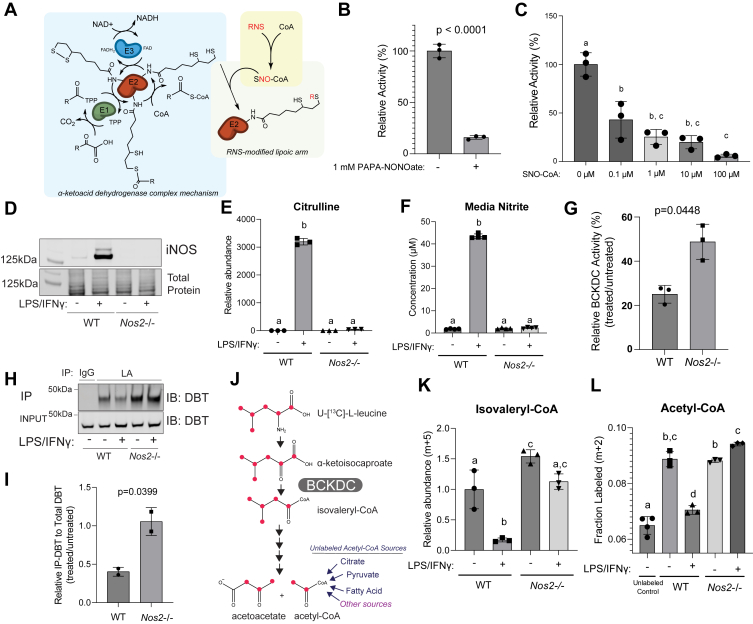
**Reactive nitrogen species inhibits BCKDC in activated macrophages.***A*, *model schematic.* RNS can cause inhibitory modification of the E2 subunit’s catalytic lipoic arm, through the targeted delivery *via* SNO-CoA. *B*, relative BCKDC activity in mitochondria lysate from WT RAW264.7 cells, after *in vitro* incubation with PAPA-NONOate (1 mM) for 5 h at room temperature. *C*, relative BCKDC activity in matrix lysate from WT RAW264.7 cells after *in vitro* incubation with varied doses of SNO-CoA in the presence of NADH (200 μM) for 3 h at room temperature. *D*–*F*, immunoblot for iNOS in whole cell lysate (*D*), relative intracellular level of citrulline (*E*), and concentration of nitrite in the spent media (*F*), of WT and *Nos2*−/− RAW264.7 cells with or without 48-h LPS/IFNγ stimulation. *G*, percent BCKDC activity in WT or *Nos2*−/− RAW264.7 cells after 48-h LPS/IFNγ stimulation normalized to unstimulated. *H* and *I*, the level of functional lipoic arm on DBT in WT or *Nos2*−/− RAW264.7 cells with or without 48-h LPS/IFNγ stimulation. *H*, representative immunoblots of DBT in RAW264.7 whole cell lysates (input) or after immunoprecipitation of lipoic acid or IgG control. *I*, relative fraction of DBT with functional lipoic arm in stimulated state compared to unstimulated state, in WT or *Nos2*−/− cells, quantified based on blots, N = 2. *J*, U-[^13^C]-L-leucine isotopic tracing schematic. *Red dots* represent ^13^C conversion through the leucine catabolic pathway. *K*, relative abundance of isovaleryl-CoA (m+5 labeled from U-[^13^C]-L-leucine) in WT or *Nos2*−/− RAW264.7 cells with or without 48-h LPS/IFNγ stimulation. *L*, the fraction of m+2–labeled acetyl-CoA after 48 h labeling in media containing U-[^13^C]-L-leucine (N = 3) or unlabeled control (N = 4) in WT or *Nos2*−/− RAW264.7 cells with or without 48-h LPS/IFNγ stimulation. *B*, *G*, and *I*, statistical analysis was performed with unpaired student’s *t* test with *p*-values reported. *C*, *E*, *F*, *K*, and *L*, statistical analysis was performed using one-way ANOVA followed by post hoc Tukey’s test. Bars with different lower-case letters (a, b, c, or d) indicate a statistically significant difference with *p* < 0.05, with exact *p*-values reported in Supporting Information. *B*, *C*, *E*–*G*, *I*, *K*, and *L*, all bars and error bars represent mean ± SD, N = 3, unless otherwise noted. BCKDC, branched chain α-ketoacid dehydrogenase complex; IFNγ, interferon-gamma; iNOS, inducible nitric oxide synthase; LPS, lipopolysaccharide; RNS, reactive nitrogen species.

Specifically, BCKDC is the rate-liming step in the oxidation of the branched chain amino acids (BCAA), leucine, isoleucine, and valine. The catabolism of these essential amino acids contributes carbon units to various metabolic pathways, including the tricarboxylic acid (TCA) cycle, ketone body metabolism, and lipid synthesis. In addition, BCAAs and their catabolic intermediates play important roles in nutrient sensing and signaling (2, 3). The first step of BCAA catabolism is mediated by branched chain aminotransferase, Bcat1 or Bcat2, which transfers the amino group from valine, isoleucine, or leucine to alpha-ketoglutarate, producing the corresponding α-ketoacids, α-ketoisovalerate, α-keto-β-methylvalerate, and α-ketoisocaproate, respectively. Next, BCKDC oxidizes these α-ketoacids to their corresponding acyl-CoA products, α-methylbutyryl-CoA, isobutyryl-CoA, and isovaleryl-CoA, respectively. BCAA oxidation flux is particularly high in skeletal muscle (4), where it plays a key role in human health (5, 6, 7). Dysregulated BCAA metabolism is associated with many diseases, including cardiovascular disease, such as atherosclerosis and heart failure, and metabolic disorders, such as diabetes mellitus and obesity (2, 8, 9, 10, 11, 12).

As BCKDC, PDHC, and OGDC control important crossroads of the metabolic network, their activities are dynamically regulated by layers of molecular mechanisms. Particularly, multiple mechanisms acting through posttranslational modifications of E1, E2, or E3 subunits of these enzymes have been found to play key roles in their regulation (13, 14, 15, 16, 17, 18, 19, 20, 21, 22, 23). Recently, we discovered that reactive nitrogen species (RNS) produced by inducible nitric oxide synthase (iNOS), encoded by the gene *Nos2*, in classically activated macrophages led to profound inhibition of PDHC and OGDC, *via* a previously unknown posttranslational modifications (24). This inhibition is caused by loss of functional lipoic arm on their E2 subunit, as can be detected by immunoblotting. However, mass spectrometry–based analysis showed this loss of functional lipoic arm is largely not due to absence of bound lipoic cofactor, rather, RNS can cause a series of covalent modifications of the bound lipoic arm, preventing it from cycling between its reduced and oxidized forms to perform its catalytic activity (Fig. 1*A*). Furthermore, we demonstrated that the RNS-driven inhibition acts through specifically modifying the active thiols of the lipoic arm, by showing inhibition of purified PDHC by RNS depends on the presence of its substrates to generate reactive thiols. Without the addition of substrates (pyruvate and CoA), E2 subunit’s lipoic arm is mainly in its oxidized form. In this condition, incubating purified PDHC with NO donor alone does not cause substantial inhibition of PDHC activity. In contrast, incubating purified PDHC with its substrates, pyruvate and CoA, or its product NADH allows the lipoic arm to convert to its reduced form *via* E1 and E2 subunit activity or reversed E3 subunit activity, respectively, exposing the reactive thiols. Consistently, incubating purified PDHC with NO donor in the presence of substrates causes substantial inactivation of PDHC. Given the mechanistic similarity among α-ketoacid dehydrogenase complexes, it is conceivable that a similar mechanism could also regulate BCKDC. Additionally, many other cell types beyond macrophages also produce nitric oxide (NO); therefore it is also likely that the RNS-driven regulation of these complexes is important in these cell types. However, these hypotheses have not been tested.

Here, through a series of *in vitro* and in cell experiments, we show that RNS can indeed inhibit BCKDC by modifying the lipoic arm on its E2 subunit. This mechanism not only inhibits BCKDC alongside PDHC and OGDC in classically activated macrophages but also in muscle cells, where BCKDC has an important role. Upon exposure to tumor necrosis factor-alpha (TNFα) and interferon-gamma (IFNγ), muscle cells express iNOS and produce NO (25). This NO production has been implicated in muscle cachexia and altered mitochondrial metabolism (26). We found that TNF-α and IFN-γ stimulated NO production in myotubes and myoblasts lead to significant rewiring of their mitochondrial metabolism and altered energy charge.

In addition to targeting E2 subunit’s lipoic arm, RNS have also been shown to inhibit α-ketoacid dehydrogenase complex through mechanisms acting on the E3 subunit (DLD). In classically activated macrophages, RNS can cause inhibitory cysteine nitrosylation of DLD (21). The normal catalytic function of DLD in α-ketoacid dehydrogenase complexes is to re-oxidize the reduced lipoic arm on E2 subunit using a cysteine-cysteine active site. Based on this close interaction between the subunits, we reasoned that the RNS-driven modifications on the E2 subunit's lipoic arm could promote the modification of E3 subunits. Indeed, here we find the RNS-driven inhibition of the E3 subunit largely depends on lipoic arm modification of the E2 subunit, providing a mechanistic link between the two recently discovered RNS-driven inhibitory mechanisms.

Together, these data demonstrate a common mechanism which allows RNS to inhibit important enzymes across the lipoic arm–dependent dehydrogenase family, including BCKDC. Such regulation by RNS has significant biological impacts in multiple cell types that are capable of producing RNS. It is likely to have broader relevance in other cell types that are influenced by RNS in the microenvironment as well.

## Results

### RNS cause strong inhibition of BCKDC

To test the hypothesis that NO can inhibit BCKDC, we first incubated mitochondrial lysate from RAW 264.7 cells with NO donor, PAPA-NONOate. Indeed, *in vitro* treatment with NO donor led to a profound inhibition of BCKDC activity (Fig. 1*B*).

We hypothesized this inhibition was mediated mainly through a mechanism similar to what we recently found with PDHC and OGDC (24): RNS cause a series of inactivating S-modifications of the lipoic arm on their E2 subunit, and such mechanism is highly specific and efficient because in cells, RNS can react with CoA to form SNO-CoA, which, *via* binding to the E2 subunit at the CoA-binding site, can deliver the modifications to the lipoic arm in a targeted manner. If the hypothesis that RNS inhibit BCKDC *via* a similar mechanism is true, we would predict that BCKDC can also be directly inhibited by SNO-CoA in the presence of NADH (to generate reduced thiol on lipoic arm) with a high potency. We therefore incubated mitochondrial lysate with varied doses of SNO-CoA in the presence of NADH and measured BCKDC activity. Indeed, SNO-CoA inhibited BCKDC in a dose-dependent manner, with concentration as low as 0.1 μM can cause over 50% activity reduction in 3 h and 10 μM near completely inactivated BCKDC (Fig. 1*C*). These results provided *in vitro* evidence that BCKDC can be efficiently inhibited by RNS similar to PDHC and OGDC.

### NO production leads to BCKDC inhibition in activated macrophages

We next tested the hypothesis that such RNS-driven inhibition of BCKDC occurs in cells. In macrophages, classical activation by lipopolysaccharide (LPS) and IFNγ induces the expression of iNOS (Fig. 1*D*), resulting in the production of NO, as indicated by the accumulation of intracellular citrulline (Fig. 1*E*) and nitrite (a final product of RNS) in media (Fig. 1*F*). In the macrophage cell line, RAW 264.7, BCKDC activity is reduced by ∼80% upon classical activation, and such activation-induced BCKDC inhibition is significantly rescued in *Nos2*−/− cells (Fig. 1*G*), showing NO as an important driver of the BCKDC inhibition in activated macrophages. To test whether this NO-dependent inhibition of BCKDC is mediated by changes in the lipoic arm, we probed for the level of functional lipoic arm on BCKDC’s E2 subunit, DBT, by immunoprecipitation. Although BCKDC activity decreased substantially upon activation, the total level of DBT in input is slightly higher upon activation in both WT and *Nos2*−/− macrophages (Fig. 1, *H* and *I*), possibly due to compensation, suggesting the activation-induced inhibition is not due to reduced DBT level but strong inactivation of its catalytic activity. Correlating with overall BCKDC activity, the level of functional lipoic arm on DBT decreased substantially upon activation in WT macrophages, but such decrease was prevented by *Nos2* knock out (Fig. 1, *H* and *I*), suggesting that NO causes changes to the lipoic arm which then mediate the BCKDC inhibition upon activation.

BCKDC is the rate-limiting step in BCAA catabolism. To examine the impact of NO production on BCAA metabolism in macrophages, we applied isotopic tracing with U-[^13^C]-L-leucine. Oxidation of U-[^13^C]-L-leucine by BCKDC produces 5-labeled isovaleryl-CoA, which can be further metabolized to labeled acetyl-CoA, whereas the unlabeled fraction of acetyl-CoA originates from other sources, including citrate, pyruvate, and β-oxidation of fatty acids (Fig. 1*J*). In WT RAW264.7 cells, stimulation by LPS and IFNγ greatly reduced the abundance of 5-labeled isovaleryl-CoA (Fig. 1*K*) and reduced the contribution of U-[^13^C]-L-leucine to acetyl-CoA production to near-background level (Here, the background was the fraction of M+2 acetyl-CoA measured in cells cultured in fully unlabeled media. The M+2 arises from natural abundance of C13 and S34) (Fig. 1*L*). Both the stimulation-induced reduction in isovaleryl-CoA abundance and in acetyl-CoA labeling from leucine were rescued by *Nos2* knock out (Fig. 1, *K* and *L*). Together, these results show NO production causes lipoic arm alteration and inhibition of BCKDC, and reduction in BCAA oxidation, in macrophages upon classical activation.

### NO inhibits α-ketoacid dehydrogenase complexes in myotubes and myoblasts

Many tissues and cell types have the capability of producing NO by nitric oxide synthases for functions including signaling, pathogen killing, and regulation of angiogenesis, vasodilation, and neural functions (27, 28, 29, 30). Additionally, cells that do not actively produce NO themselves can be impacted by RNS in the microenvironment generated by neighboring cells. Therefore, this mechanism for RNS to inhibit of α-ketoacid dehydrogenase complexes is likely to have broad biological significance in a variety of cellular systems beyond macrophages. Skeletal muscle is known to express iNOS to produce NO and have elevated RNS upon stimulation with the cytokines (25, 31, 32, 33). We hypothesized that α-ketoacid dehydrogenase complexes are inhibited by RNS in muscle cells upon cytokine stimulation and tested this hypothesis using a widely used cell model, C2C12 myoblast cells, which can be differentiated to myotubes (34, 35, 36).

**Figure 2:**
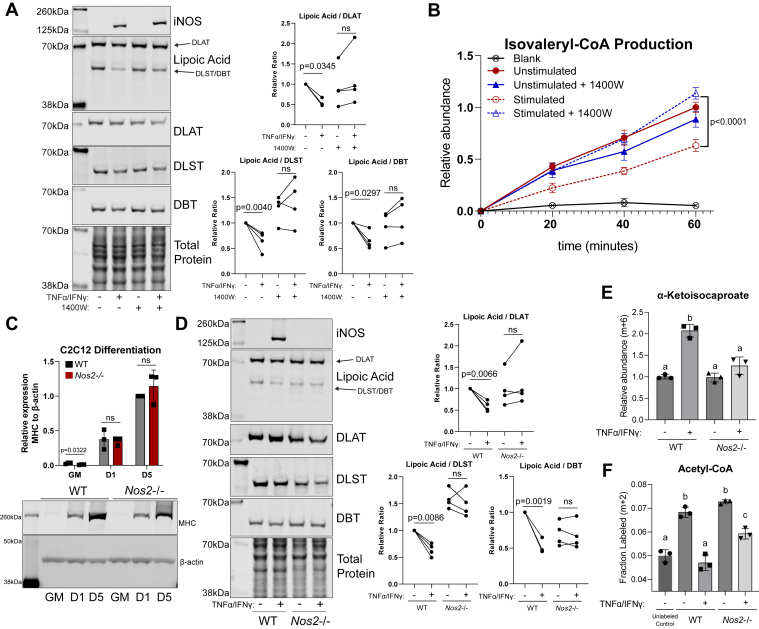
**Nitric oxide production inhibits BCKDC in C2C12 myotubes.***A*, representative immunoblots for iNOS, DLAT, DLST, DBT, and lipoic moiety in whole cell lysates of WT C2C12 myotubes with or without 48-h TNFα/IFNγ stimulation and with or without treatment with 1400W (200 μM). This experiment was repeated four times. To compare the changes in functional lipoic arm upon stimulation, the relative ratio of lipoic band to its corresponding total E2 subunit band (DLAT for the lipoic band at ∼70 kDa and DLST or DBT for the lipoic band at ∼50 kDa) was quantified and normalized to unstimulated, untreated condition. Each dot represents quantified results from one independent experiment. Statistical analysis for significance was performed with paired student’s *t* test with *p*-values reported. ns indicates *p* > 0.05. *B*, relative BCKDC activity as measured by isovaleryl-CoA production for α-ketoisocaproate over time in crude mitochondria isolation from WT C2C12 myotubes treated with or without 1400W (200 μM) and with or without 48-h TNFα/IFNγ stimulation. *C*, representative immunoblot for myosin heavy chain (MHC) and beta-actin over a time course as WT or *Nos2−/−* C2C12 cells differentiation. GM, undifferentiated C2C12 cells cultured in growth media (GM); D1, first day in differentiation media; D5, after 5 days in differentiation media. This experiment was repeated with three replicates, with quantification of relative MHC expression normalized to beta-actin expression presented in bar graph. *D*, the same as (*A*) but with genetic *Nos2* KO instead of 1400W treatment. *E*, relative abundance of α-ketoisocaproate (6-labeled from U-[^13^C]-L-leucine) in WT or *Nos2*−/− C2C12 myotubes with or without 48-h TNFα/IFNγ stimulation. *F*, the fraction of m+2–labeled acetyl-CoA after 48 h labeling with U-[^13^C]-L-leucine in WT or *Nos2*−/− C2C12 myotubes with or without 48-h TNFα/IFNγ stimulation. *B* and *C*, statistical analysis for significance was performed with unpaired student’s *t* test with *p*-values reported. ns indicates *p* > 0.05. *E* and *F*, statistical analysis for significance was performed with one-way ANOVA followed by a post hoc Tukey’s test. Bars with different lower-case letters (a, b, or c) indicate a statistically significant difference with *p* < 0.05, with exact *p*-values reported in Supporting Information. *A*–*F*, all bars and error bars represent mean ± SD. N = 3, unless otherwise noted. BCKDC, branched chain α-ketoacid dehydrogenase complex; DBT, dihydrolipoamide branched chain transacylase; DLAT, dihydrolipoamide S-acetyltransferase; DLST, dihydrolipoamide S-succinyltransferase; IFNγ, interferon-gamma; iNOS, inducible nitric oxide synthase; TNFα, tumor necrosis factor-alpha.

In differentiated C2C12 myotubes stimulated with TNF-α and IFN-γ for 48 h, iNOS expression is induced, and the levels of functional lipoic arm in both the bands corresponding to the molecular weight of DLAT (E2 subunit of PDHC, ∼70 kDa) and DLST or DBT (E2 subunit of OGDC and BCKDC, respectively, both ∼ 50 kDa) are reduced relative to total level of their corresponding E2 subunits (Fig. 2*A*). Treating cells with a selective inhibitor of iNOS, N-(3-(aminomethyl) benzyl)-acetamidine (1400W), rescued the stimulation-induced decrease in the functional lipoic arm level relative to E2 subunits (Fig. 2*A*). Consistent with the changes in lipoic arm, BCKDC activity, as measured in isolated mitochondria lysate, is reduced by ∼50% upon stimulation, and this inhibition is rescued by iNOS inhibition (Fig. 2*B*).

We further tested the effect of NO on the changes in the lipoic arm of α-ketoacid dehydrogenase complexes using a genetic knock out of *Nos2*. Like WT C2C12 cells, *Nos2* KO cells can be sufficiently differentiated to myotubes, as indicated by the great increase of myosin expression after differentiation (Fig. 2*C*). Similar to what is observed with pharmacological inhibition of iNOS, the stimulation-induced decrease of functional lipoic arm relative to total DLAT, DLST, and DBT level was prevented in *Nos2*−/− myotubes (Fig. 2*D*).

To further probe BCKDC activity in cells, we performed U-[^13^C]-L-leucine tracing. Upon stimulation with TNF-α and IFN-γ, cellular level of labeled α-ketoisocaproate, the substrate of BCKDC, accumulated in WT myotubes but not *Nos2*−/− myotubes (Fig. 2*E*). The fraction of 2-labeled acetyl-CoA decreased to blank level upon stimulation in WT myotubes, and this loss of labeling from U-[^13^C]-L-leucine is significantly reversed by *Nos2*−/− (Fig. 2*F*). This NO-dependent buildup of substrate and decrease in labeling incorporation into downstream metabolite indicated NO inhibits BCKDC activity in cells.

**Figure 3:**
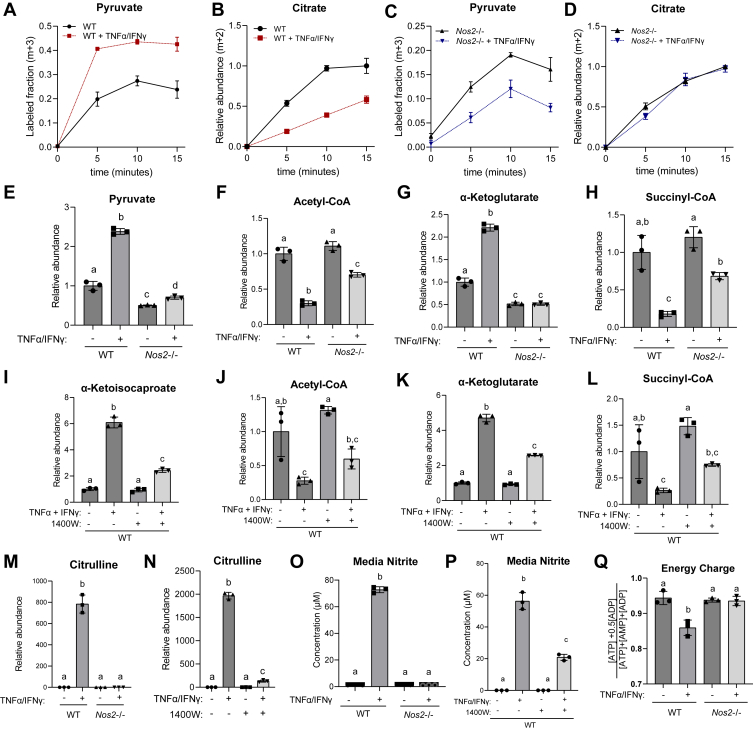
**Nitric oxide production causes inhibition of PDHC and OGDC in C2C12 myotubes.***A* and *B*, kinetic labeling incorporation from U-[^13^C]-D-glucose to (*A*) pyruvate (m+3 labeled) and (*B*) citrate (m+2) in WT C2C12 myotubes with or without 48-h TNFα/IFNγ stimulation. *C* and *D*, same as (*A*) and (*B*), except with *Nos2*−/− C2C12 myotubes. Each time point and error bars represent mean ± SD, N = 3. *E*–*H*, relative abundance of intracellular pyruvate (*E*), acetyl-CoA (*F*), α-ketoglutarate (*G*), and succinyl-CoA (*H*) in WT or *Nos2*−/− C2C12 myotubes with or without 48-h TNFα/IFNγ stimulation. *I*–*L*, relative abundance of intracellular α-ketoisocaproate (*I*), acetyl-CoA (*J*), α-ketoglutarate (*K*), and succinyl-CoA (*L*) in WT C2C12 myotubes with or without 1400W (200 μM) treatment, with or without 48-h TNFα/IFNγ stimulation. *M* and *N*, relative abundance of citrulline in WT or Nos2−/− C2C12 myotubes (*M*), or WT myotubes with or without 1400W (200 μM) (*N*), with or without 48-h TNFα/IFNγ stimulation. *O* and *P*, nitrite concentration in spent media from WT or Nos2−/− C2C12 myotubes (O) or WT myotubes with or without 1400W (200 μM) (*P*). *Q*, energy charge of WT or *Nos2*−/− C2C12 myotubes with or without 48-h TNFα/IFNγ stimulation. *E*–*Q*, statistical analysis was performed with one-way ANOVA followed by a post hoc Tukey’s test. Bars with different lower-case letters (a, b, or c) indicate a statistically significant difference with *p* < 0.05, with exact *p*-values reported in Supporting Information. All bars and error bars represent mean ± SD, N = 3. IFNγ, interferon-gamma; TNFα, tumor necrosis factor-alpha.

Based on the observed changes in the lipoic arm (Fig. 2, *A* and *D*), we also expected PDHC and OGDC activity to be inhibited in an NO-dependent manner in TNF-α– and IFN-γ–stimulated myotubes. To examine PDHC activity and glucose oxidation in myotubes, we performed kinetic labeling with U-[^13^C]-glucose tracer. U-[^13^C]-glucose is quickly converted to U-[^13^C]-pyruvate *via* glycolysis in both stimulated and unstimulated condition (Fig. 3*A*). The labeled pyruvate can be metabolized by PDHC to 2-labeled acetyl-CoA, which is then converted to 2-labeled citrate. In WT myotubes, TNF-α and IFN-γ stimulation caused the labeling incorporation from glucose into citrate to be much slower (Fig. 3*B*), even though the labeling incorporation into pyruvate is higher (Fig. 3*A*), suggesting greatly reduced flux through PDHC. In contrast, this reduced rate of labeling incorporation from pyruvate into citrate was not observed when *Nos2*−/− myotubes were activated (Fig. 3, *C* and *D*). These results suggest NO production inhibits intracellular PDHC activity and glucose oxidation in activated myotubes. Consistently, we observed substantial accumulation of pyruvate, the substrate of PDHC, and depletion of acetyl-CoA, the product of PDHC, in stimulated WT myotubes, and these stimulation-induced changes are largely prevented by *Nos2*−/− (Fig. 3, *E* and *F*). Similarly, the substrate for OGDC, α-ketoglutarate, accumulated significantly, and the product of OGDC, succinyl-CoA, depleted upon stimulation in WT myotubes, but these changes are significantly rescued in *Nos2*−/− myotubes (Fig. 3, *G* and *H*).

Similar to genetic *Nos2* knock out, treating cells with iNOS inhibitor 1400W also partially reversed the stimulation-induced accumulation of α-ketoacids and depletion of succinyl-CoA and acetyl-CoA (Fig. 3, *I*–*L*). However, the rescue was weaker than the genetic knock out. This correlates with the fact that genetic *Nos2* KO completely ablated NO production but 1400W treatment did so incompletely, as evidenced by measurements of intracellular citrulline, a product of iNOS (Fig. 3, *M* and *N*), and extracellular nitrite (Fig. 3, *O* and *P*), suggesting possible dose-dependent effect of NO.

As the results above suggested NO production causes inhibition of all three α-ketoacid dehydrogenase complexes, PDHC, OGDC and BCKDC, which control the mitochondrial oxidation of important nutrients—glucose, glutamine, and BCAA, respectively—such inhibition can have significant impact on cellular bioenergetics. Indeed, consistent with a previous report (26), we observed cellular energy charges significantly decreased upon stimulation of myotubes, and this decrease is prevented by *Nos2*−/− (Fig. 3*Q*).

**Figure 4:**
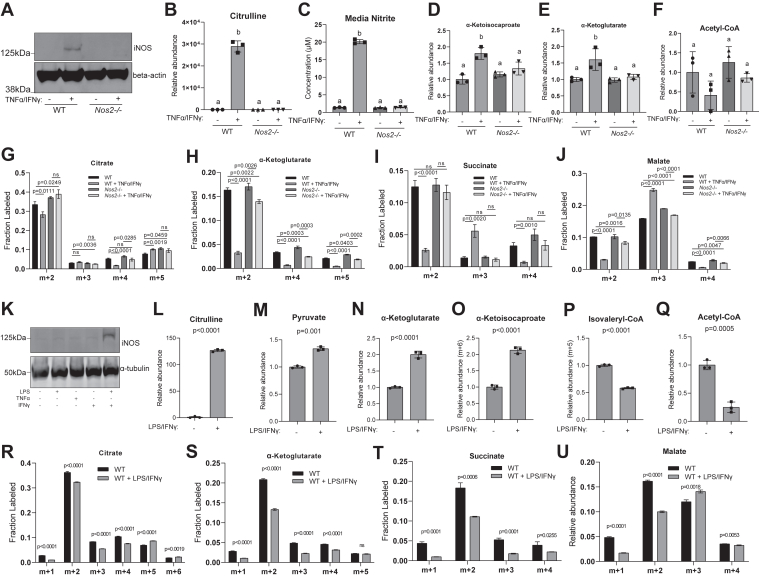
**Nitric oxide inhibits α-ketoacid dehydrogenase complexes in undifferentiated C2C12 myoblasts.***A*, immunoblot for iNOS and beta-actin in whole cell lysate of WT or *Nos2*−/− C2C12 myoblasts with or without 48 h TNFα/IFNγ stimulation. *B* and *C*, relative abundance of intracellular citrulline (*B*) normalized to unstimulated and extracellular nitrite concentration in spent media (*C*), in WT or *Nos2*−/− C2C12 myoblasts with or without 48-h TNFα/IFNγ stimulation. *D*–*F*, relative abundance of intracellular α-ketoisocaproate (*D*), alpha-ketoglutarate (*E*), acetyl-CoA (*F*) in WT or *Nos2*−/− C2C12 myoblasts with or without 48-h TNFα/IFNγ stimulation. *G*–*J*, labeling patterns of citrate (*G*), α-ketoglutarate (*H*), succinate (*I*), and malate (*J*) in unstimulated or 48-h TNFα/IFNγ–stimulated C2C12 myoblasts labeled in media containing U-[^13^C]-D-glucose for 24 h. *K*, immunoblot for iNOS and alpha tubulin in whole cell lysate of WT C2C12 myoblasts upon 24-h stimulation with indicated combination of stimuli. *L*–*N*, relative abundance of citrulline (*L*), pyruvate (*M*), and α-ketoglutarate (*N*) in WT C2C12 myoblasts with or without 24-h LPS/IFNγ stimulation. *O* and *P*, relative abundance of α-ketoisocaproate (6-labeled from U-[^13^C]-L-leucine) (*O*) and isovaleryl-CoA (5-labeled from U-[^13^C]-L-leucine) (*P*) in WT C2C12 myoblasts with or without 24-h LPS/IFNγ stimulation, cultured in media containing U-[^13^C]-L-leucine for 24 h. *Q*, relative abundance of acetyl-CoA in WT C2C12 myoblasts with or without 24-h LPS/IFNγ stimulation. *R*–*U*, labeling patterns of citrate (*R*), α-ketoglutarate (*S*), succinate (*T*), and malate (*U*) in unstimulated or 24-h LPS/IFNγ–stimulated WT C2C12 myoblasts labeled in media containing U-[^13^C]-D-glucose for 24 h. *B*–*F*, statistical analysis was performed with one-way ANOVA followed by a post hoc Tukey’s test. Bars with different lower-case letters (a or b) indicate a statistically significant difference with *p* < 0.05, with exact *p*-values reported in Supporting Information. *G*–*J* and *L*–*U*, statistical analysis for significance was performed with unpaired student’s *t* test with *p*-values reported. ns indicates *p* > 0.05. *B*–*J* and *L*–*U*, all bars and error bars represent mean ± SD, N = 3. IFNγ, interferon-gamma; iNOS, inducible nitric oxide synthase; LPS, lipopolysaccharide; TNFα, tumor necrosis factor-alpha.

Like myotubes, when undifferentiated myoblasts are stimulated with TNF-α and IFN-γ, they express iNOS (Fig. 4*A*) and produce NO as well, as indicated by the accumulation of intracellular citrulline (Fig. 4*B*) and nitrite in media (Fig. 4*C*). Consistent with NO driving inhibition of α-ketoacid dehydrogenases, we found the level of α-ketoacids accumulate, and acetyl-CoA decreased upon TNF-α and IFN-γ stimulation in myoblasts, and such changes are rescued by *Nos2* knock out (Fig. 4, *D*–*F*). Incorporation of labeled glucose into TCA cycle intermediates is also reduced upon stimulation, which is rescued by *Nos2* knock out (Fig. 4, *G*–*J*). Similarly, iNOS expression and activity can also be induced in myoblasts by LPS and IFN-γ stimulation (Fig. 4, *K* and *L*). And we observed similar metabolic alterations, including accumulation of the substrates of α-ketoacid dehydrogenases (Fig. 4, *M*–*O*), decrease of their corresponding acyl-CoAs (Fig. 4, *P* and *Q*), and reduced labeling from glucose into TCA cycle intermediates (Fig. 4, *R*–*U*), upon LPS and IFN-γ stimulation. In summary, in all the tested conditions when NO production is activated, cellular metabolism is significantly altered by NO-driven inhibition of α-ketoacid dehydrogenases, in both myotubes and myoblasts.

### RNS-driven lipoic modification on E2 subunit promotes E3 subunit inhibition

The α-ketoacid dehydrogenase complexes are subjected to the regulation by many mechanisms. Besides the inhibitory modifications of the lipoic arm on E2 subunit, it has been recently discovered that in macrophages, RNS can also inhibit PDHC *via* another mechanism—cysteine nitrosylation on its E3 subunit (DLD) (21). The normal catalytic function of DLD is to use a cysteine-cysteine active site to re-oxidize the reduced lipoic arm on E2 subunit, then transfer the electron to FAD, then NAD, to produce NADH (Fig. 1*A*). Based on this close interaction between E2 and E3 subunits, we hypothesized that the RNS-driven lipoic modification on E2 subunit can further promote the cysteine modification on E3 subunit through mechanisms such as trans-nitrosylation.

**Figure 5:**
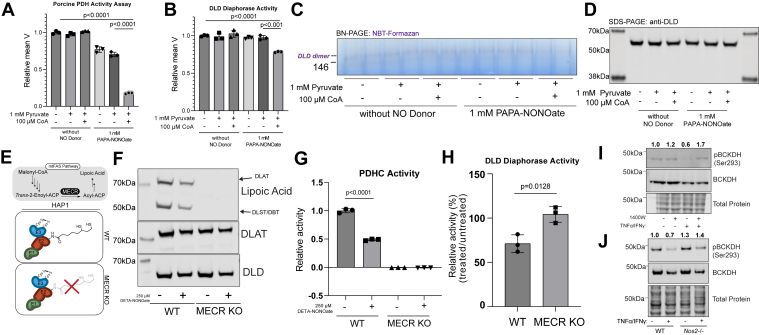
**Reactive nitrogen species–driven lipoic modification on E2 subunit promotes E3 subunit inhibition.***A*, relative enzymatic activity of purified porcine PDHC after incubation at room temperature with or without PAPA-NONOate (1 mM), in the presence or absence of pyruvate (1 mM) and CoA (100 μM). *B*, DLD activity measured by spectrometric assay after purified porcine PDHC was incubated with indicated combination of PAPA-NONOate (1 mM), pyruvate (1 mM), and CoA (100 μM). *C*, in-gel assay after purified porcine PDHC was incubated with indicated combination of PAPA-NONOate (1 mM), pyruvate (1 mM), and CoA (100 μM). DLD activity is indicated by purple dye intensity at the molecular weight of DLD dimer (∼146 kDa) in native gel. Samples loaded on gel in paired technical duplicates *D*, immunoblot for total DLD level in the same samples used in the PDHC, spectrometric, and in-gel activity assays in (*A*), (*B*), and (*C*), respectively. *E*, schematic of WT and MECR KO HAP1 cells. *F*, immunoblot for lipoic acid, DLAT, and DLD from WT and MECR (KO) HAP1 cells with or without 250 μM DETA-NONOate treatment. *G*, relative enzymatic activity of PDHC from WT or MECR KO HAP1 cells with or without 250 μM DETA-NONOate treatment. *H*, percent DLD enzymatic activity in WT or MECR KO HAP1 cells treated with 250 μM DETA-NONOate normalized to respective untreated condition. *I*, immunoblots for phosphorylated (Ser293) and total BCKDH in unstimulated or stimulated (TNFα and IFNγ, 48 h) WT C2C12 myotubes with or without treatment of 1400W. *J*, immunoblots for phosphorylated (Ser293) and total BCKDH in unstimulated or stimulated (TNFα and IFNγ, 48 h) WT (WT) or *Nos2−/−* C2C12 myotubes. *I* and *J*, relative ratio of phospho-BCKDH (Ser293) to total-BCKDH (normalized to WT unstimulated) reported above respective blot. *A*, *B*, *G*, and *H*, statistical analysis for significance was performed with unpaired student’s *t* test with *p*-values reported. All bars and error bars represent mean ± SD, N = 3. DLAT, dihydrolipoamide S-acetyltransferase; DLD, dihydrolipoamide dehydrogenase; IFNγ, interferon-gamma; MECR, mitochondrial trans-2-enoyl-CoA reductase; PDHC, pyruvate dehydrogenase complex; TNFα, tumor necrosis factor-alpha.

To test this hypothesis, we took advantage of the fact that, as demonstrated in our previous work (24), the modification of E2 subunit’s lipoic arm by RNS depends on the presence of substrates (pyruvate and CoA) to generate reduced thiols that is susceptible to modifications and to deliver RNS modifications to lipoic arm *via* SNO-CoA (Fig. 1*A*). If the modification and inhibition of DLD is resulted from the interaction with E2 subunit’s modified lipoic arm, it too would be dependent on the presence of substrates. We therefore incubated purified PDHC with NO donor, PAPA-NONOate, in the presence or absence or pyruvate and CoA. Only when both pyruvate and CoA are present, did the NO donor cause a large reduction in overall PDHC activity (Fig. 5*A*) and in specific DLD activity as measured by spectrometric assay (Fig. 5*B*) and in-gel activity assay (Fig. 5, *C* and *D*) following previously established protocols (21, 37, 38, 39). The observation that NO donor can cause significant inhibition of DLD activity without changing total DLD level in the presence of pyruvate and CoA (Fig. 5, *B*–*D*) confirmed that in normal cellular environment, where pyruvate and CoA are present, production of NO would be capable of modifying and inhibiting DLD, as recently reported in activated macrophages (21). However, the inhibition of DLD activity (18%, Fig. 5*B*) is relatively small compared to the inhibition of overall PDHC activity (82%, Fig. 5*A*), suggesting the contribution of E3 inhibition is minor and E2 subunit inhibition is the major driver of overall PDHC inhibition by RNS. The fact that without pyruvate or CoA, NO donor alone does not cause significant DLD inhibition demonstrated that NO does not directly cause inhibitory modifications of DLD, instead, the inhibition is mediated by the interaction between E3 subunit and E2 subunit with RNS-modified lipoic arm.

To further test the dependence of E3 subunit inhibition on E2 subunit’s lipoic arm in cells, we used a cell model that lacks lipoic arm on α-ketoacid dehydrogenase complexes’ E2 subunit. Mitochondrial trans-2-enoyl-CoA reductase (MECR), a required enzyme in the lipoic acid biosynthetic pathway (Fig. 5*E*), was knocked out in HAP1 cell line. As expected, MECR-null cells have no detectable level of lipoic arm on DLAT, DLST, or DBT, while WT HAP1 cells do (Fig. 5*F*). We then treated WT or MECR KO cells with NO donor. The level of functional lipoic arm on α-ketoacid dehydrogenase complexes’ E2 subunits reduced upon NO donor treatment (Fig. 5*F*), and consistently, overall PDHC activity is reduced in WT HAP1 cells (Fig. 5*G*). MECR KO cells have no overall PDHC activity (Fig. 5*G*), as expected from the lack of lipoic arm. Importantly, when we specifically measured the activity of DLD, we found that DLD activity was only significantly decreased after NO donor treatment in WT, but not MECR KO cells (Fig. 5*H*). This result provided in-cell evidence that the inhibition of DLD by RNS depends on the lipoic arm on E2 subunit.

Another well-known posttranslational modification mechanism to regulate the activity of some α-ketoacid dehydrogenase complexes acts through their E1 subunits. The E1 subunits of PDHC and BCKDC can be phosphorylated at specific sites. These phosphorylation sites inhibit PDHC and BCKDC activity and are under the regulation of specific kinases and phosphatases (13, 16, 40, 41). OGDC, however, has not been shown to be regulated by similar E1 phosphorylation. Interestingly, we found that in myotubes, BCKDH phosphorylation decreases upon stimulation with cytokines (Fig. 5, *I* and *J*), which can counteract the NO-dependent E2 inactivation and potentially reduce overall activity loss. When stimulated cells were treated with iNOS inhibitor or when *Nos2* was knocked out, in addition to the rescue of functional lipoic arm (Fig. 2, *A* and *D*), we observed the stimulation-induced decrease in E1 subunit phosphorylation was also reversed (Fig. 5, *I* and *J*). These results suggested that changes in BCKDC E1 phosphorylation and E2 lipoic modification have compensatory effect during myotube activation by cytokines. However, the appeared compensatory relationship E2 subunit modification by RNS and E1 subunit phosphorylation are condition specific, as we have observed in macrophages upon classical activation, inhibitory PDHC E1 subunit phosphorylation is increased at the same time E2 subunit’s lipoic arm is inactivated by RNS (42). This condition-specific relationship reflects that the phosphorylation of E1 subunit and the RNS-driven E2 subunit modifications occur through independent mechanisms, in contrast to the inhibitory modifications of E2 and E3 subunits by RNS are mechanistically connected.

## Discussion

Mitochondrial α-ketoacid dehydrogenase complexes catalyze crucial reactions at the crossroads within the metabolic network. Here we demonstrate that NO can strongly inhibit BCKDC. This study, together with our recently published work, revealed that RNS are capable of broadly inhibiting all the α-ketoacid dehydrogenase complexes through a common mechanism—modifying and inactivating the catalytic lipoic arm of their E2 subunits. We demonstrated that this mechanism drives significant alterations in the metabolism of carbohydrates and amino acids across multiple cell types, including macrophages, myotubes, and myoblasts, under conditions in which NO production is induced. It has been previously reported that NO production in cytokine-activated muscle cells has important physiological effects, including impairment of myoblast proliferation and differentiation (43) and induction of apoptosis in aging-induced sarcopenia (44). The inhibition of α-ketoacid dehydrogenase complexes, and the resulting alterations in mitochondrial metabolism, can play a role in mediating these effects. Given that purposeful production of NO by iNOS, eNOS (primarily expressed in endothelial cells), and nNOS (primarily expressed in neurons), as well as the generation of RNS as metabolic by-products, occurs in many physiological and pathological contexts (28, 45); this mechanism is likely to have broad significance in regulating metabolism. Alterations of metabolism by RNS have the potential to have numerous downstream impacts *via* mechanisms including affecting protein acetylation and succinylation by altering acetyl-CoA and succinyl-CoA availability or changing cellular energetic status. The broad downstream effects and their mechanisms remain to be further examined.

The α-ketoacid dehydrogenase complexes are subjected to tight regulation by a variety of mechanisms. Here we investigated the relationship between the RNS-driven inhibitory modifications on the E2 subunit and other regulatory posttranslational modifications targeting α-ketoacid dehydrogenase complexes’ E3 or E1 subunits. We found that RNS-driven modification of the E2 subunit’s lipoic arm promotes inhibition of the E3 subunit. This molecular connection has important implications in the specificity of RNS-driven DLD inhibition and in the extent and reversibility of overall α-ketoacid dehydrogenase inhibition by RNS. In high RNS conditions, such as classically activated macrophages, we found that α-ketoacid dehydrogenases are specifically and profoundly inhibited, while cell viability and the activity of many other mitochondrial enzymes remains high (42). If RNS cause inhibitory DLD nitrosylation by direct, nonenzymatic interaction with DLD, it is mechanistically unclear why DLD would be preferably modified and inhibited, over many other mitochondrial proteins that have cysteine residues which can potentially be modified. This specificity question is explained by our model of multistep modification transfer: In cells, RNS can react with CoA, a relatively abundant thiol-containing metabolite, and generate SNO-CoA. Through the specific binding of SNO-CoA to the E2 subunit, the modification is efficiently delivered to the thiol of lipoic arm; and through the local interaction between the E2 and E3 subunits, the E3 subunit is further modified. Through this mechanism, both E2 and E3 subunits can be inactivated by RNS, causing greater effect on overall α-ketoacid dehydrogenase inhibition. As a result, to recover the overall enzyme activity, the inhibitory modifications on both E2 and E3 subunits need to be removed. The reversibility under specific cellular conditions is an important direction for future studies.

Overall, this study extended our knowledge about the mechanisms impacting the activity of α-ketoacid dehydrogenase complexes. This work showed that strong inhibition of α-ketoacid dehydrogenase complexes by RNS can have significant effects in cellular metabolism across various cell types. These findings merit future investigation to examine the broader physiological or pathological effects of this mechanism *in vivo* and explore the translational implications in conditions where elevated RNS play a key role, such as inflammatory disorders and cardiovascular diseases.

## Experimental procedures

### Cell culture

RAW 264.7 cells, WT or *Nos2*−/−, were cultured at 37 °C with 5% CO_2_ in RPMI 1640 media with 1% penicillin-streptomycin, 25 mM Hepes, and 10% fetal bovine serum (FBS). Dialyzed fetal bovine serum was used in the place of FBS for metabolomics and isotope-tracing experiments. Media was replaced every 24 h. To stimulate the cells, 50 ng/ml LPS (*E. coli* 0111:B4) (Sigma-Aldrich, L3024) and 10 ng/ml recombinant mouse interferon-γ (IFN-γ) (R&D Systems, 485-MI-100) were added to the media and maintained with subsequent media change.

Murine myoblast cell line, C2C12 (ATCC), were cultured in growth media (Dulbecco’s modified Eagle’s medium (DMEM) with 10% FBS and 1% penicillin-streptomycin) at 37 °C with 5% CO_2_. To differentiate C2C12 cells to myotubes, media was replaced with differentiation media (DMEM with 2% donor Equine serum (Hyclone) and 1% penicillin-streptomycin) once cells reached 70 to 90% confluency. Media was replaced every 48 h for 5 days (*initial 24 h in differentiation media is termed ‘D0’, and then D1, D2, D3, D4, and D5 each subsequent 24 h*). After differentiation, media was changed to DMEM with 2% FBS or dialyzed fetal bovine serum (for metabolomic or isotopic tracing experiments), and media was refreshed every 24 h. For cytokine stimulation, 20 ng/ml recombinant mouse TNF-α (R&D Systems, 41-0MT0-25CF) and 12 ng/ml recombinant mouse IFN-γ were added to the media.

The human chronic myeloid leukemia haploid cell line, HAP1, WT or MECR KO (generated by Horizon, HZGHC006857c007), were cultured in Improved modified Eagle’s medium with 10% FBS and 1% penicillin-streptomycin at 37 °C with 5% CO_2_. For NO donor treatment, 250 μM of DETA-NONOate was added to the media for 48 h with media and donor replacement every 24 h.

All cell lines were tested for *mycoplasma* contamination.

For leucine tracing experiments, media without L-leucine was supplemented with U-[^13^C]-L-leucine (Cambridge Isotope Laboratories, CLM-2262-H) at formulation concentration and was used in the place of chemically identical regular unlabeled media. Both RAW264.7 and C2C12 cells were cultured with stable isotope for 48 h with media changes at 24 and 2 h prior to metabolite extraction. For kinetic glucose tracing, media without D-glucose supplemented with U-[^13^C]-D-glucose (Cambridge Isotope Laboratories, CLM-1396-1) at formulation concentration was used.

For iNOS inhibitor treatment of C2C12 myotubes, 200 μM 1400W (Cayman Chemical, 81520) was added to the media 24 h prior to experiment start as pretreatment. The inhibitor was maintained in the media at the same concentration throughout the experiment duration.

### CRISPR-Cas9–based genetic knockout of Nos2

*Nos2*−/− KO cells were generated as previously described (24). Briefly, 2 × 10^6^ C2C12 myoblast cells were transfected *via* electroporation with 1 μM fluorescent transactivating CRISPR RNA (tracerRNA, ITT, catalog no. ATTO550), 1 μM RNA targeting mouse *Nos2* (crRNA, GTGACGGCAAACATGACTTC, IDT Design ID: Mm.Cas9.NOS2.1.AA), and 1 μM HiFi Cas9 enzyme (IDT, catalog no. 1081060) in 100 μl Nucleofector solution V plus supplement (Lonza, catalog no. VCA-1003), using the preprogrammed electroporation protocol B-032 on Nucleofector II/2b. Immediately, cells were plated on a 35-mm plate with DMEM media with 10% FBS without penicillin/streptomycin. Eighteen hours after transfection, cells positive for fluorescent tracrRNA were single-cell sorted by FACS (BD FACSAria III) onto a 96-well plate in DMEM media with 10% FBS and 1% penicillin/streptomycin. Single-cell colonies were expanded and subsequently screened *via* Western blot for the lack of iNOS expression after 48 h stimulation with TNFα and IFNγ. Further validation of positive hits (myoblasts without iNOS expression after cytokine treatment) was performed by differentiating the selected myoblast clones into myotubes followed by 48-h treatment with TNFα and IFNγ and subsequent Western blot for iNOS expression, measurement of nitrite concentration in the media using Griess Reagent System (Promega, G2930), and measurement of intracellular citrulline abundance by LCMS.

### Protein extraction, SDS-PAGE, and immunoblotting

Whole cell lysate was collected using RIPA buffer (150 mM NaCl, 1% NP-40 substitute, 50 mM Tris, 0.4 mM EDTA, 0.1% SDS, 0.5% sodium deoxycholate, 10% (v/v) glycerol, pH = 8.0). Lysate was incubated on ice for 15 min and spun at 12,000*g* for 5 min at 4 °C. Total soluble protein concentration in supernatant was determined with bicinchoninic acid (BCA) assay (Thermo Fisher Scientific, Pierce 23225). Denatured gel was run using a Thermofisher Scientific Mini Gel Tank system with Bolt Bis-Tris 8% or 4 to 12% gels and Bolt MES or MOPS running buffer. Proteins were then transferred to nitrocellulose membrane using Bolt Transfer Buffer. Total protein stain was used (Li-COR, 926-11011) to visualize loading. Membranes were blocked in 5% nonfat dairy milk in Tris-Buffer Saline with 0.01% Tween-20 (0.01% TBS-T) for 1 h at room temperature. Primary antibodies were diluted 1:1000 in 5% bovine serum albumin in 0.01% TBS-T and incubated with membrane overnight at 4 °C. Membranes were washed with TBS-T and placed with secondary antibody diluted 1:10,000 in 5% bovine serum albumin in TBS-T for 1 h at room temperature. Blots were imaged on Odyssey CLx Imaging System (LI-COR Biosciences).

For immunoblotting, the following antibodies were used: anti-DLST (Abcam, ab187699), anti-DBT (Abcam, ab151991), anti-lipoic acid (Millipore Sigma, 43-769-5100UL), anti-DLAT (Abcam, ab172617), anti-phospho-BCKDH-E1α (Ser293) (Cell Signaling Technology, 40368S), anti-BCKDH-E1α (Cell Signaling Technology, 90198S), anti-iNOS (Cell Signaling Technology, 2982S), anti-DLD (Abcam, 133551), anti-alpha-tubulin (Cell Signaling Technology, 3873S), anti-beta-actin (Cell Signaling Technology, 4967S), anti-Myosin Heavy Chain (Abcam, ab37484), IRDye 800 CW Goat Anti-Mouse IgG Secondary (LI-COR, 926-32210), and IRDye 800 CW Goat Anti-Rabbit IgG Secondary (LI-COR, 926-32211). Antibodies were validated by respective vendors.

### Immunoprecipitation

WT or *Nos2*−/− RAW264.7 cells were collected into centrifuge tubes after washing twice with Dulbecco’s phosphate buffer saline (D-PBS) and scrapping off the culture plates. Cell suspensions were spun at 500*g* for 10 min at 4 °C and supernatant was discarded. Protein was extracted from the cell pellets using extraction buffer (20 mM Tris HCl, pH 8.0, 150 mM NaCl, 1 mM EDTA, 1 mM EGTA, 1% NP-40, and Pierce protease and phosphatase inhibitors (Thermo Fisher Scientific, A32957 and A32953)) at a volume of 0.350 ml per 3.00 × 10^7^ cells. Cell mixtures were incubated on ice for 30 min, then centrifuged at 5000*g* for 10 min at 4 °C. Supernatant was transferred to a tube, and total soluble protein concentration was determined with BCA assay. Dynabeads Protein G (Thermo Fisher Scientific, 10003D) (200 μl per isolation) were washed three times with citrate-phosphate buffer (470 mg citric acid, 920 mg dibasic sodium phosphate dihydrate, pH = 8.0), then incubated with 2 μl of anti-lipoic acid antibody, or nonspecific IgG control, in 200 μl D-PBS with gentle mixing for 40 min at room temperature and washed three times with citrate-phosphate buffer. Antibody was cross-linked by washing twice with 0.2 M triethanolamine (pH = 8.2) and resuspending in fresh DMP solution (20 mM dimethyl pimelimidate dihydrochloride (Thermo Fisher Scientific, P08925G) in 0.2 M triethanolamine, pH = 8.2 (DOT Scientific Inc, DST23040-0.1)) and incubating for 30 min at room temperature. The reaction was terminated by resuspending beads in 50 mM Tris buffer (pH = 7.5) and incubating for 15 min. Cross-linked beads were washed three times with PBS with 0.1% Tween-20. To immunoprecipitate protein containing lipoic moiety, 2 mg of whole cell lysate were mixed with cross-linked bead–Ig complex and the mixture was rotated end-over-end overnight at 4 °C. Then the beads were washed three times with PBS and protein was eluted with 50 μl of glycine elution buffer (50 mM glycine, pH = 2.8). To ensure complete elution, an additional 30 μl of elution buffer was added to the beads, and the eluants were pooled. The eluant was then neutralized with 1:1 (v/v) neutralization buffer (1 M Tris, pH = 7.5). Total protein concentration of eluant was determined with BCA assay, then the same amount was loaded for immunoblotting for DBT.

### Metabolite extraction and LCMS analysis

Cells were washed three times with D-PBS, and intracellular metabolites were extracted with cold 80:20 methanol:H_2_O (v/v, LCMS grade). Samples were dried under nitrogen gas and resuspended in LCMS-grade H_2_O.

Samples were analyzed using a Thermo Q-Exactive mass spectrometer coupled to a Vanquish Horizon UHPLC. Analytes were separated on a 100 × 2.1 mm, 1.7 μM Acquity UPLC BEH C18 Column (Waters), with a gradient of solvent A (97:3 H_2_O:methanol, 10 mM TBA, 9 mM acetate, pH 8.2) and solvent B (100% methanol) at 0.2 ml/min flow rate. The gradient is as follows: 0 min, 5% B; 2.5 min, 5% B; 17 min, 95% B; 21 min, 95% B; 21.5 min, 5%. Data were collected in full-scan negative mode. Setting for the ion source were as follows: 10 aux gas flow rate, 35 sheath gas flow rate, two sweep gas flow rate, 3.2 kV spray voltage, 320 °C capillary temperature, and 300 °C heater temperature.

The metabolites reported were identified based on exact *m/z* and retention times determined with chemical standards. Data were analyzed with MAVEN (46, 47).

To quantify changes in relative metabolite levels, metabolite abundance measured by ion count in LCMS analysis were normalized to total protein content. To quantify absolute abundance for AMP, ADP, and ATP to calculate cellular energy charge, the ion count measured by LCMS was converted to molar quantity based on calibration curves obtained by running various concentrations of purified AMP, ADP, and ATP chemical standards on LCMS using the same method. Energy charge is calculated based on Equation 1.

(1) $$Energy\;Charge=\frac{\left[ATP\right]+0.5\left[ADP\right]}{\left[ATP\right]+\left[AMP\right]+\left[ADP\right]}$$

### Crude mitochondria isolation and matrix enrichment

Mitochondria isolation and matrix enrichment was performed as previously described (21, 48, 49) with adaptations. Briefly, cells were harvested from tissue culture plate after washing three times with D-PBS. Cells were pelleted by spinning at 1000*g* for 5 min at 4 °C. Cell pellet was resuspended in isolation buffer (10 mM Tris-MOPS, pH = 7.4, 1 mM EGTA/Tris, and 200 mM sucrose) and then homogenized with Teflon pestle operating at 1600 rpm with 50 passes. Homogenate was spun at 600*g* for 10 min at 4 °C. Supernatant was transferred to a new tube and spun at 7000*g* for 10 min at 4 °C. Pellet was resuspended in isolation buffer then spun at 7000*g*. Mitochondria pellet was lysed with hypotonic lysis buffer (20 mM Tris, pH 7.4, 1 mM EDTA, and Pierce protease and phosphatase inhibitors) and incubated on ice for 15 min. For crude mitochondria use, mitochondria protein concentration is determined with BCA assay.

For matrix lysate enrichment, pelleted crude mitochondria were lysed with 150 μl hypotonic lysis buffer, sonicated on ice for 5 s followed by 30 s rest for a total of four times at 40% amplitude with a probe sonicator. After the addition of 30 μl 1 M NaCl and 20 μl 50% glycerol (v/v) to reach final concentration of 150 mM NaCl and 5% glycerol, lysate was spun at 20,000*g* for 30 min at 4 °C. For DLD activity assays, matrix lysate was dialyzed (3.5 K MWCO membrane) overnight at 4 °C rocking in 20 mM sodium phosphate buffer. The protein concentration in supernatant matrix lysate was determined with BCA assay.

### Branched chain α-ketoacid dehydrogenase activity assay

BCKDC activity assay in lysate was performed as previously described with adaptations (31). To initiate reaction, 200 μl of assay buffer with substrate mixture (30 mM K_3_PO_4_, 2 mM MgSO_4_, 2 mM DTT (Thermo Fisher Scientific, AAJ1539706), 0.56 mM TPP (Sigma-Aldrich, C8754-1G), 0.56 mM CoA (Cayman Chemical, 16147), 1 mM NAD+ (Cayman Chemical, 16077), Triton X-100, 0.2 mM alpha-ketoisocaproate (Cayman Chemical, 21052-5), and 5 μM rotenone (VWR, 10189-314) at pH=7.0), which was prewarmed to 30 °C, and 20 μg of crude isolated mitochondria or matrix lysate, as specified in figure legends, were mixed in each well of 96-well plate. Reaction was allowed to proceed at 30 °C. At designated time points (typically every 20 min from reaction start to 1 h), 50 μl or 60 μl of reaction mixture sample was collected and quenched in 4× volume (200 μl or 240 μl) of LCMS-grade methanol. Samples were spun and supernatant were dried under nitrogen gas, then resuspended in LCMS-grade H_2_O and analyzed by LCMS. The reaction rate was quantified by the production of isovaleryl-CoA over time. The slope was fitted by linear regression. As a blank control, the same amount of mitochondria lysate was mixed with assay buffer without alpha-ketoisocaproate.

For experiments involving *in vitro* treatment of mitochondria lysates with NO donor or SNO-CoA, lysate was diluted to 1.5 μg/μl in assay buffer (30 mM K_3_PO_4_, pH 7.0); PAPA-NONOate or SNO-CoA were added and incubated at room temperature for 3 to 5 h, as specified in figure legend. SNO-CoA was prepared as previously described (24, 50, 51) by combining 100 mM CoA in 1 M HCl with 100 mM NaNO_2_ in 100 μM EDTA and 100 μM DPTA in a 1:1 (v/v) ratio.

### Purified porcine PDHC activity assay

The PDHC activity assay was performed as previously described with adaptations (24). Briefly, purified porcine PDHC (Sigma-Aldrich, P7032-10UN) (0.7 units/ml) was incubated at room temperature for 18 h with NO Donor, PAPA-NONOate (1 mM) (Cayman Chemical, 82410), with indicated combinations of CoA (100 μM) and pyruvate (1 mM) in 20 mM sodium phosphate buffer (pH 7.2). After incubation, the protein mixture was diluted 1:20 in assay solution containing thiamine pyrophosphate (100 μM), CoA (2 mM), pyruvate (2 mM), and NAD+ (10 mM) in 20 mM sodium phosphate buffer (pH 7.2). PDHC activity was quantified by the rate of NADH production, as measured by the increasing NADH absorbance at 340 nm over time using a BioTek Epoch2 microplate reader. Absorbance was measured continuously, and the mean velocity was determined from the linear portion of the curve. Data was analyzed using Gen5 TS v.2.09 software (https://www.agilent.com/en/product/microplate-instrumentation/microplate-instrumentation-control-analysis-software/imager-reader-control-analysis-software/biotek-gen5-software-for-detection-1623227) (BioTek Instruments, Inc).

### Cell lysate PDHC activity assay

PDHC activity in cell lysate from HAP1 cells was analyzed using a pyruvate dehydrogenase enzyme activity microplate assay kit (Abcam, ab109902) per the manufacturer’s instructions. This kit measures PDHC activity by monitoring pyruvate-dependent NADH production. The NADH level was measured by absorbance of NADH-coupled dye (450 nm) using a BioTek Epoch2 plate reader. Data were analyzed using Gen5 TS v.2.09 software (BioTek Instruments, Inc).

### Blue native-PAGE and DLD activity assay

For native gel analysis, the Novex Native Bis-Tris Gel System was used (Thermo Fisher Scientific). Samples were loaded in 4 to 16% Bis-Tris NativePAGE gel and run at 150 V for 1 h at 4 °C with anode buffer in outer chamber and light blue cathode buffer in inner chamber. After 1 h, the light blue cathode buffer was replaced with anode buffer. The gel was run for an additional 1 h at 250 V on ice at 4 °C.

In gel, DLD activity assay was performed as previously described (21, 37, 38). Briefly, native gel was immediately removed from cassette and incubated in activity assay buffer (50 mM potassium phosphate, pH = 7.0, 0.2 mg/ml nitro blue tetrazolium (NBT) chloride (Alta Aesar, B23792.02), and 0.1 mg/ml NADH (Cayman Chemical, 16078)) for 40 to 50 min until purple bands appeared. The gel was then imaged on an EPSON Scan V700. After the image was obtained, gel was fixed, stained with Coomassie R-250, and destained for visualization of the protein standard (Thermo Fisher Scientific, LC0725). The product of the diaphorase activity of DLD is NBT-formazan, which has a maximum absorbance between 500 to 600 nm (39). Therefore, DLD activity was quantified by the rate of diaphorase activity as measured by the increasing NBT-formazan absorbance at 568 nm over time using a BioTek Epoch2 microplate reader. Absorbance was measured continuously, and the mean velocity was determined from the linear portion of the curve. As a blank control, the same amount of mitochondria lysate was mixed with assay buffer without NADH. Mean velocity was normalized to relative DLD protein expression by corresponding Western blot. Data was analyzed using Gen5 TS v.2.09 software (BioTek Instruments, Inc).

### Measurement of nitrite concentration

To measure nitrite production by cells, 2 ml media was incubated with each well of cells in 6-well plate (macrophages or myotubes) or 10 ml media was incubated with each 10 cm plate of cells (macrophages), for 48 h, then spent media was collected. Nitrite concentration in spent media was measured using Griess Reagent System (Promega, G2930) per manufacturer’s instructions.

### Statistical analysis

Unless otherwise stated in figure legend, for comparisons between two groups, a nonpaired students’ *t* test was performed. For comparisons between three groups or more, one-way ANOVA followed by Tukey’s post hoc test for multiple comparisons was performed.

### Software

LCMS data analysis was performed with Maven Version 6.2. Immunoblots were visualized and quantified using Image Studio Lite version 5.2 for Windows (LI-COR Biosciences). Data were graphed, and all statistical analyses were completed in GraphPad Prism (https://www.graphpad.com/) version 9.4.1 for Windows (Graph Pad Software). Figures were created with Adobe Illustrator 2023 and ChemDraw, Professional, Version 22.0.0.22.

## Data availability

All data are contained within the article. Source data for all the figures are available upon request to the corresponding author.

## Supporting information

This article contains supporting information.

## Conflict of interest

The authors declare that they have no conflicts of interest with the contents of this article.
